# Antiretroviral Intensification and Valproic Acid Lack Sustained Effect on Residual HIV-1 Viremia or Resting CD4+ Cell Infection

**DOI:** 10.1371/journal.pone.0009390

**Published:** 2010-02-23

**Authors:** Nancie M. Archin, Manzoor Cheema, Daniel Parker, Ann Wiegand, Ronald J. Bosch, John M. Coffin, Joseph Eron, Myron Cohen, David M. Margolis

**Affiliations:** 1 University of North Carolina at Chapel Hill, Chapel Hill, North Carolina, United States of America; 2 HIV Drug Resistance Program, National Cancer Institute, National Institutes of Health, Frederick, Maryland, United States of America; 3 Harvard School of Public Health, Boston, Massachusetts, United States of America; Karolinska Institutet, Sweden

## Abstract

**Background:**

Human immunodeficiency virus (HIV) infection that persists despite antiretroviral therapy (ART) is a daunting problem. Given the limited evidence that resting CD4+ T cell infection (RCI) is affected by the histone deacetylase (HDAC) inhibitor valproic acid (VPA), we measured the stability of RCI and residual viremia in patients who added VPA with or without raltegravir (RAL), or enfuvirtide (ENF) with or without VPA, to standard ART.

**Methods:**

Patients with plasma HIV RNA<50 c/mL added sustained-release VPA (Depakote ER®) twice daily, RAL 400 mg twice daily, or ENF 90 mcg twice daily. Change in RCI was measured by outgrowth assays. Low-level viremia was quantitated by single-copy plasma HIV RNA assay (SCA).

**Results:**

In three patients on standard ART a depletion of RCI was observed after 16 weeks of VPA, but this effect waned over up to 96 weeks of further VPA. In two patients ENF added to stable ART had no effect on RCI. Simultaneous intensification with ENF and addition of VPA had no effect on RCI frequency in one patient, and resulted in a 46% decline in a second. No significant depletion of RCI (>50%) was seen in six volunteers after the addition of RAL and VPA. In 4 of the 6 patients this lack of effect might be attributed to intermittent viremia, low VPA levels, or intermittent study therapy adherence. Overall, there was no effect of the addition of RAL or ENF on low-level viremia measured by SCA.

**Conclusions:**

The prospective addition of VPA and RAL, VPA and ENF, or ENF failed to progressively reduce the frequency of RCI, or ablate intermittent and low-level viremia. New approaches such as more potent HDAC inhibition, alone or in combination with intensified ART or other agents that may disrupt proviral latency must be pursued.

## Introduction

Given the costs and difficulties in maintaining suppression of viremia in the HIV-infected patients for decades, and the challenges that face effective prevention of HIV infection, interest has reawakened in interventions that might eradicate HIV infection [1]. Currently, two challenges to the eradication of infection are apparent: the persistence of latent infection, and the persistent expression of low levels of virions from cells infected prior to ART initiation. Clearance of HIV infection will almost certainly require a multimodal approach that includes potent suppression of HIV replication, therapies that reach all compartments of residual HIV expression, and depletion of any reservoirs of persistent, quiescent proviral infection. Reagents that selectively induce the expression of quiescent proviral genomes but have limited effects on host cell activation and susceptibility to infection might allow outgrowth of latent HIV, and avoid the pitfalls of global T cell activation [2], [3].

Histone deacetylase (HDAC) is a critical regulator of HIV latency [4], and in ex vivo assays HDAC inhibition leads to HIV outgrowth from the resting CD4+ T-cells of aviremic patients [3]. In one small pilot study RCI was depleted when intensified ART was given in combination with generic VPA, an anticonvulsant and non-selective HDAC inhibitor [5]. However, little effect was seen in two observational studies and one prospective trial in which VPA was given without intensified ART [6]–[8].

In this prospective trial [8] a decrease in RCI was observed in only 4 of the 11 patients. To extend these observations, we measured the stability of RCI and of low-level viremia in patients on standard ART after prolonged treatment with sustained-release VPA and ART, and after intensified of ART by an agent acting via a novel mechanism of action, entry inhibition or integrase inhibition.

## Methods

HIV-infected volunteers receiving stable ART with plasma HIV-1 RNA<50 copies/ml and a CD4 count of >300/µl for at least 6 months were studied. 3 HIV-infected males stably suppressed on ART and previously treated with VPA (patients 1–3, as reported in ref. 8) in whom a significant decline in RCI was measured after 16 weeks of VPA agreed to further study on treatment with VPA. As reported [8], leukapheresis was performed on two occasions (day −49 to −27 and day 0) prior to, and after 12 and 16 weeks of protocol therapy with the sustained-release formulation of VPA (Depakote ER®). Patients 1–3 had a depletion of RCI measured at weeks 12–16. As per protocol, VPA therapy was continued past week 16 and repeat studies were planned at weeks 48 and 96. Patient 3 was assessed at week 32, as he declared his intention to leave the study after 48 weeks.

2 HIV-infected males stably suppressed on ART in whom no effect of VPA was measured (patients 6 and 9, as reported in ref. 8) agreed to intensify baseline ART with ENF. When RCI was unchanged after ENF, patients 6 agreed to later intensify baseline ART with ENF and VPA simultaneously. One newly enrolled HIV-infected male stably suppressed on ART without previously VPA exposure (patient 12) simultaneously added VPA and ENF to baseline ART. As previously reported, patients 6 and 9 stopped VPA when no decline of RCI was measured, but continued baseline ART [8]. As per protocol, an additional RCI measurement was made at study re-entry, before initiation of ENF 90 mcg twice daily. Again patients stopped ENF when no decline of RCI was measured, but continued baseline ART [8]. Patients 6 later agreed to re-enter study, simultaneously adding VPA and ENF to baseline ART. Patient 12 initiated study therapy with VPA and ENF, but used the generic immediate-release formulation of VPA.

In a parallel study, seven HIV-infected males, stably suppressed on ART (patients 13–18) enrolled to study the effect of the receipt of VPA and RAL. Baseline ART included nucleoside RT inhibitors and protease inhibitors (7 patients), or non-nucleoside RT inhibitors (5 patients). CD4 cell counts at entry ranged from 320 to 1242 cells/µl (17–42%). Study therapy for this parallel protocol included RAL 400 mg twice daily and VPA (Depakote ER®) 500–750 mg bid for patients 13–18.

Enrollment criteria for both protocols have been described [8]. All patients provided written informed consent, and the study was approved by the University of North Carolina Office of Human Research Ethics Institutional Review Board.

Lymphocytes were obtained by continuous-flow leukapheresis. Isolation of resting CD4+ T cells, recovery and quantification of replication competent virus was performed as described, with inter-assay variance of 0.3 log [8]. By protocol, changes in RCI of more than 50% were deemed significant. Flow cytometry analysis was performed on lymphocytes isolated at leukapheresis as described [5] to verify purity of resting CD4+ T cell isolation.

Plasma HIV-1 RNA concentrations were measured by Roche Amplicor (Roche Molecular Systems, Branchburg, NJ) with an assay detection limit of 50 copies/ml. To accurately measure the extent of low-level viremia, an ultra-sensitive, quantitative real-time, RT-PCR single copy assay (SCA) capable of detecting and quantifying plasma HIV-1 RNA to a limit of detection of 1 copy per ml was performed [9]. SCA was performed on plasma samples collected at the screening visit, at 27–49 days before the start of protocol therapy, on the day 0 and weeks 1, 2, 4, 8, 12 and 16 after the start of protocol therapy.

## Results

Overall, VPA therapy was generally well tolerated, with only minor or transient adverse events not clearly related to VPA. VPA levels were infrequently above 60 mcg/ml. RAL was well tolerated without adverse events. ENF was well tolerated, with minor, transient injection site reaction in one patient. No significant adverse effects (greater than ACTG Grade II) were noted in any patients.

Patients 1–3 were found to have a >50% depletion of RCI on two resting cell assays at week 12 and 16 of VPA therapy in a study reported previously [ref. 8]. They agreed to extended VPA therapy for re-evaluation at weeks 48 and 96. Despite continuous ART and VPA, the depletion of RCI seen at week 16 regressed to baseline at 48 and 96 weeks (Table 1). SCA measurements generally correlated with concurrent Amplicor results (Table 1). Intermittent viremia was detected in patient 2 by Amplicor assay, but never on days when SCA was measured. In patients 1–3 SCA viremia was rarely >1 copy/ml. Total VPA levels were measured near trough, prior to daily dosing, and ranged from undetectable to 82 mcg/ml.

**Table 1:** Effect of VPA and intensified ART on resting cell infection and low-level viremia.

Patient	Infected Resting CD4+ Cells per Billion*					Mean VPA (mcg/ml)	Amplicor HIV RNA copies/ml	Median HIV SCA** (copies/ml)
	baseline ART	Weeks of ART & VPA						
		16	32	48	96			
**1**	187	90	n.d.	170	219	44 (33–57)	<50 (n = 10)	<1 (<1 to 1; n = 9)
**2**	52	22	n.d.	82	96	53 (40–74)	<50 (n = 9) and 56, 100	<1 (n = 4)
**3**	70	22	57	73	n.d.§	50 (<12–82)	<50 (n = 8)	<1 (n = 4)
	**ART**		**ART & ENF×16 wks**					
**9**	4254		4177			n.a.	<50 (n = 6) and 75	13 (2 to 24; n = 6)
**6**	59		116			n.a.	<50 (n = 8)	<1 (n = 6)
	**ART**		**ART & ENF & VPA×16 wks**					
**6**	109		127			61.4 (23–83)	<50 (n = 6)	<1 (<1 to 1; n = 9)
**12**	175		95			68†† (55–90)	<50 (n = 7)	<1 (<1; n = 5)
	**ART**		**ART & RAL & VPA×16 wks**					
**13**	500		340			45† (24–64)	<50 (n = 12) and 118, 75, 73, 90	9 (2–24; n = 11)
**14**	210		250††			36† (20–60)	<50 (n = 13) and 102	1 (<1–18; n = 12)
**15**	410		600			42 (23–51)	<50 (n = 10) and 60, 73, 98, 192	3 (<1–8; n = 5)
**16**	400		440			59 (46–75)	<50 (n = 13)	1 (<1 to 7; n = 13)
**17**	27		33			70 (62–79)	<50 (n = 14)	<1 (<1 to 35; n = 12)
**18**	620		650			77 (50–93)	<50 (n = 12)	<1 (<1 to 1.8; n = 11)

*All results represent pooled assays at entry/week −4, and week 12/16. Baseline ART assays for patients 6, 9, and 12 represent pooled assays from entry/week −4 and 2 prior time points. Weeks 32, 48, 96 are assays from only those time points.

**Simultaneous Amplicor assays at all SCA time points were <50 copies, except for patient 3 at day of study entry when Amplicor = 58 and SCA>1000.

†Declined VPA dose escalation.

††Intermittent non-adherence to study medication.

§Early study discontinuation.

Patients 6 and 9 in the same prior study [8] had no decline RCI after 16 weeks of VPA. Nine and six months (respectively) after VPA discontinuation, these patients intensified continuous ART with the addition of ENF. 16 weeks of ENF had no effect on the frequency of RCI (Table 1). In patient 9 but not patient 6, intermittent viremia of >50 copies/ml by Amplicor assay was observed. In this patient SCA was routinely positive at 2–24 copies/ml. These results appear consistent with variation in PCR-based detection of stable low-level viremia, as described in a longitudinal study [10].

Four months later patient 6, in whom RCI had been unaffected by the addition of either VPA or ENF, agreed to undergo simultaneous intensification with ENF and the reinitiation of VPA. After 16 weeks of ART, VPA, and ENF, RCI remained low but stable. VPA levels were adequate, and SCA stably <1 copy/ml.

The use of the sustained-release formulation of VPA (Depakote ER®) in these patients and those previously described [8] could have resulted in a blunting of peak VPA levels needed for sufficient HDAC inhibition in vivo. Therefore in an additional volunteer (patient 12) naïve to VPA and ENF, we studied the effect of simultaneous ENF intensification and generic VPA twice daily. SCA was stably <1 copy/ml, and VPA levels adequate, but we discovered the patient had not taken 20–30% of study medication at the end of the study period. Nevertheless, we observe a 46% decline in RCI, a trend that failed to achieve our pre-established criterion of a significant decline (>50%).

Patients 13–18, stably suppressed on ART, intensified ART with the addition of 400 mg RAL twice daily and simultaneously added VPA. Surprisingly, despite RAL intensification, patients 13, 14, and 15 had episodic viremia detected by Amplicor. Patients 13 and 15 had four episodes of detectable viremia by Amplicor assay, while patient 14 was >50 copies on one occasion. VPA dose was increased to 1500 mg/day in patients 15 and 17 for trough levels consistently below 40 mcg/ml; levels of >50 mcg/ml were then measured in patient 17, but not in patient 15. In patients 13, 14, and 15, VPA levels were suboptimal, and patients 13 and 14 were unwilling to escalate the dose of drug. Patient 14 missed at least a week of study drug while traveling. None of patients 13–18 had significant declines (>50%) in RCI. Overall, the addition of RAL or ENF had no effect on viremia measured by SCA.

## Discussion

In an initial study of the effect of 16 weeks of VPA prospectively added to stable ART [8], RCI declined more than 50% in 4 of 11 study subjects. While this decline might represent assay variation, it is important to note that an *increase of RCI* of more than 50% was not seen in any subjects. As assay variation is expected to be bimodal, this suggests the declines observed were biological. Further, we have not observed a significant (>50%) upward fluctuation of the RCI assay in any of the 22 patients we have intensively studied [5], [8, this report], except for subsequent assays in patients 1, 2, and 3 following the >50% decline of RCI.

In this follow-up study, depletion of RCI was not sustained in three patients studied over 48–96 weeks. It is therefore likely that the initial decline of RCI and its eventual return to baseline represents a biological event, rather than an assay artifact. A mechanistic hypothesis to explain the transient decline in RCI observed is that a population of latently HIV-infected cells are affected by VPA exposure, but that homeostatic forces later restore the size of the infected resting cell pool, as recently suggested by Chomont and colleagues [11].

Low-level viral expression as measured by SCA was not significantly diminished by VPA therapy in combination with either ENF or RAL, or by ENF alone. ENF intensification has recently been reported to have no effect on RCI, measured by similar outgrowth [12]. Just as the intensification of ART by protease inhibitors or RAL has not been observed to diminish viremia measured by SCA [13], [14], SCA was little affected by the addition of VPA and RAL, VPA and ENF, or ENF alone.

Anecdotally, it is of interest to note that the level of residual viremia appears to be related to the frequency of RCI. Given the small sample size this can only be a preliminary observation, but is consistent with the hypothesis that residual viremia originates from persistently infected, long-lived T cells.

These findings provide several important insights for future study of persistent HIV infection, and approaches to attack it. Despite a sound scientific rationale for the testing of HDAC inhibitors, only a single weak inhibitor, VPA, has been tested, and only in a handful of patients. Furthermore, since 2005 VPA donated for research use has been in a sustained-release formulation, designed to blunt the exposure of patients to higher peak serum levels of VPA. Future studies of HDAC inhibitors may seek to maximize peak drug exposure levels and measure biological markers such as acetylation of nucleosomal histone via chromatin immunoprecipitation assays. One of the patients reported herein (patient 12) was treated with generic VPA, with a modest depletion of RCI that did not achieve the pre-determined standard for significance.

Further, many patients had persistent or intermittent viremia detected by standard Amplicor assay or by SCA. Such viremia may blunt the ability to deplete RCI or to measure a depletion of RCI. The persistence of this viremia despite novel ART highlights a second significant obstacle to the eradication of HIV infection.

Depletion of RCI in the majority of HIV-infected patients with stable suppression of viremia is an unrealized goal. Vorinostat, a more potent HDAC inhibitor, can induce expression of latent HIV [15], [16] and HDAC inhibitors selective for the HDAC isoforms most relevant to HIV regulation may be more efficacious [17], [18]. Alternatively or additionally, combinatorial approaches that affect both histone acetylation and DNA/histone methylation, or NF-κB signaling and histone acetylation might be necessary to sufficiently perturb latent HIV infection [4], [19], [20]. Finally, additional approaches may be needed to ablate low-level viremia.
